# Silicon-Mediated Enhancement of Herbivore Resistance in Agricultural Crops

**DOI:** 10.3389/fpls.2021.631824

**Published:** 2021-02-11

**Authors:** Flor E. Acevedo, Michelle Peiffer, Swayamjit Ray, Ching-Wen Tan, Gary W. Felton

**Affiliations:** Department of Entomology, Pennsylvania State University, University Park, PA, United States

**Keywords:** silicon, plant resistance, plant defenses, *Spodoptera frugiperda*, tomato, maize, soybean

## Abstract

Silicon (Si) is a beneficial mineral that enhances plant protection against abiotic and biotic stresses, including insect herbivores. Si increases mechanical and biochemical defenses in a variety of plant species. However, the use of Si in agriculture remains poorly adopted despite its widely documented benefits in plant health. In this study, we tested the effect of Si supplementation on the induction of plant resistance against a chewing herbivore in crops with differential ability to accumulate this element. Our model system comprised the generalist herbivore fall armyworm (FAW) *Spodoptera frugiperda* and three economically important plant species with differential ability to uptake silicon: tomato (non-Si accumulator), soybean, and maize (Si-accumulators). We investigated the effects of Si supply and insect herbivory on the induction of physical and biochemical plant defenses, and herbivore growth using potted plants in greenhouse conditions. Herbivory and Si supply increased peroxidase (POX) activity and trichome density in tomato, and the concentration of phenolics in soybean. Si supplementation increased leaf Si concentration in all plants. Previous herbivory affected FAW larval weight gain in all plants tested, and the Si treatment further reduced weight gain of larvae fed on Si accumulator plants. Notably, our results strongly suggest that non-glandular trichomes are important reservoirs of Si in maize and may increase plant resistance to chewing herbivores. We conclude that Si offers transient resistance to FAW in soybean, and a more lasting resistance in maize. Si supply is a promising strategy in management programs of chewing herbivores in Si-accumulator plants.

## Introduction

Silicon (Si), one of the most abundant elements on earth is ubiquitously present in the soil, but mainly in forms unavailable for plant uptake (Tubaña and Heckman, 2015). Si in the form of silicic acid is absorbed by a diverse number of plant families and stored as hydrated silica (SiO_2_nH_2_O) in roots and shoots (Hodson et al., 2005; Trembath-Reichert et al., 2015). All rooted plants interact with Si in the soil, but there is great variation in their ability to accumulate Si in their tissues. The concentration of Si in shoots can range from 0.1 to 10% of dry weight among different plant species (Ma and Yamaji, 2015). A large number of studies have reported the benefits of Si in alleviating the effects of a stunning number of plant biotic and abiotic stresses, including drought, salinity, metal toxicity, nutrient deficiency, heat, cold, pathogens, and insect herbivores (Cooke and Leishman, 2016; Imtiaz et al., 2016; Debona et al., 2017; Luyckx et al., 2017; Etesami and Jeong, 2018; Hall et al., 2019; Zhu et al., 2019; Singh et al., 2020; Vaculík et al., 2020). Evidence that Si ameliorates diverse stresses in plants led to its classification as a beneficial substance by the International Plant Nutrition Institute (IPNI) in 2015 (http://www.ipni.net/publication/nutrifacts-na.nsf/0/A7B4AB4D35C153BF85257ECE006E0E34/$FILE/NutriFacts-NA-14.pdf). However, Si is still considered a non-essential element because it is not involved in plant metabolism (Vaculík et al., 2020). Despite the documented benefits of Si in plants, its use in agriculture remains poorly adopted.

Based on their ability to accumulate Si, plants have been empirically classified as low, intermediate and high Si accumulators. However, this classification has changed due to the discovery of Si transporters that provided a better understanding of the mechanisms by which plants accumulate Si (Ma et al., 2007). Si uptake by the roots is mediated by aquaporin-like channels named LSi (Ma et al., 2007; Gaur et al., 2020). In rice, LSi1 is involved in the passive transport of Si from the soil to the root plant cells; the element is then moved inside the plant by the efflux transporter LSi2 and deposited in the form of silica in various plant structures (Ma et al., 2007; Ma and Yamaji, 2015). Furthermore, there is a specific number and amino acid sequences in aquaporin proteins necessary for Si permeability and plant uptake (Deshmukh et al., 2015). Based on these discoveries, Deshmukh et al. (2015) proposed a new classification of plants as either Si accumulators or Si excluders. More recently, Coskun et al. (2019) proposed that plants could be classified as accumulators or non-accumulators based on the presence of Si-specific aquaporin channels with functional Si permeability (Mitani and Ma, 2005; Deshmukh et al., 2015; Ma and Yamaji, 2015). The physical deposition of Si in plants has been linked with protection against insects and pathogens (Singh et al., 2020). However, Si supplementation also enhances physical and biochemical defenses in Si-accumulators and non-Si-accumulator plants (Alhousari and Greger, 2018; Singh et al., 2020).

Plants have a variety of defense mechanisms to protect themselves against herbivores; these defenses are classified as physical or chemical, which can be constitutive or inducible (War et al., 2018). Physical defenses are trichomes, thorns, lignin, waxes tough leaves, laticifers (latex), and mineral depositions (War et al., 2012). Chemical defenses include secondary metabolites (e.g., terpenes, phenols, alkaloids, sulfur, and nitrogen-containing compounds), antinutritional proteins, and enzymes (polyphenol oxidase, peroxidase, protease inhibitors, etc) (Mithöfer and Boland, 2012). Plant physical and chemical defenses may be constitutively expressed or induced by herbivory (War et al., 2018). Herbivore-induced defenses begin with the plant's recognition of damage, herbivore, and/or microbe-associated molecular patterns (DAMPS, HAMPS, MAMPS, respectively) followed by the activation of downstream reactions that occur within hours of initial herbivory (Fürstenberg-Hägg et al., 2013; Santamaria et al., 2018). Early recognition events lead to the biosynthesis of plant hormones that may include jasmonic acid (JA), salicylic acid (SA), and/or ethylene (ET), and the upregulation of gene transcripts involved in the synthesis of defensive compounds (Santamaria et al., 2018). The expression of late defense genes coding for proteinase inhibitors is activated by hydrogen peroxide and the antinutritional products are accumulated in plant tissues within days of initial herbivore damage (Fürstenberg-Hägg et al., 2013). Yet another set of late defense responses are changes in trichome density and leaf mineral accumulation that often occur in newly grown leaves several days or weeks after herbivory (Massey et al., 2007; Dalin et al., 2008). However, studies on the model grass, *Brachypodium distachyon* suggest that Si deposition in leaves starts as early as 6 h upon treatment with methyl jasmonate (Waterman et al., 2020). Plant defenses can affect herbivores directly by compromising their survival, growth, development, reproduction, oviposition, and behavior, etc. (Howe and Schaller, 2008). Biochemical defenses in the form of volatile blends and extrafloral nectar can also affect herbivores indirectly by attracting natural enemies (Karban, 2011; War et al., 2018).

Silica depositions increase the strength and rigidity of plant tissues and constitute a physical barrier for insect feeding (Bowen et al., 1992; Massey et al., 2007; Ma and Yamaji, 2015). Si accumulation enhances the abrasiveness of plant tissues causing wear of insect mouthparts and may reduce food intake and digestibility (Massey and Hartley, 2009; Leroy et al., 2019). Si can also increase callose deposition reducing food intake by sucking insects (Hao et al., 2008; Yang et al., 2018). In addition to the mechanical protection, upon damage, Si may enhance host plant resistance by increasing production of secondary metabolites and defensive proteins and enzymes in some plant species (Reynolds et al., 2016; Alhousari and Greger, 2018). Si also modifies the production of plant volatiles and enhances recruitment of herbivores' natural enemies (Kvedaras et al., 2010; Liu et al., 2017; Leroy et al., 2019). Insects exposed to Si-treated plants have exhibited reduced growth and development (Frew et al., 2017; Yang et al., 2018), alterations of feeding behavior (Assis et al., 2013), changes in oviposition preference (Peixoto et al., 2011), increased mortality (Han et al., 2015), lower fecundity (Alvarenga et al., 2017), and increased susceptibility to insecticides (Wang et al., 2020).

Induced defenses are key in host plant resistance against herbivores (Karban, 2011) but often have a metabolic cost. Plants may allocate resources to defenses that would otherwise be used for growth and other processes (Cipollini et al., 2014). It is known that Si enhances herbivore-induced defenses in different plant species, but the majority of studies have been done in wild and cultivated Poaceae plants (Alhousari and Greger, 2018; Singh et al., 2020). Moreover, most studies have measured defense responses at a single time point. It is currently unknown if the plant defense boots mediated by Si supplementation is transient or long-lasting. The temporality of plant defense induction has implications for plant resistance and possibly fitness due to the metabolic cost of those defenses. In this study, we investigated the effects of Si supplementation and insect herbivory on the induction of physical and biochemical plant defenses, foliar Si accumulation, and herbivore growth at early and late time points after initial herbivore exposure. We hypothesized that (a) Si supplementation enhances herbivore-induced defenses and foliar Si deposition in crops that accumulate different amounts of Si; (b) the timing and duration of herbivore-induced plant defenses varies for different plant species; and (c) herbivore growth is reduced when feeding on Si-supplemented plants previously exposed to herbivores. To test these hypotheses, we used the generalist herbivore fall armyworm (FAW) *Spodoptera frugiperda* and three crop species with differential ability to uptake Si: tomato (non-Si accumulator), soybean, and maize (Si-accumulators) as our model system. The results of this study contribute to a better understanding of the beneficial effects of Si in plant protection.

## Materials and Methods

### Plants

Tomato (*Solanum lycopersicum*, cv Better boy), and soybean (*Glycine max* cv *FS HiSOY*® HS33A14-98SB132B) plants were grown in Promix potting soil (Premier Horticulture, Quaker-town, PA, U.S.A.). Seeds were first germinated and then transplanted into individual 10 cm square pots (Dillen, Griffin Greenhouse Supplies, Morgantown PA, USA). Each plant was fertilized once with 3 g of the slow-release fertilizer Osmocote plus (15–9-12, Scotts, Marysville, OH, USA) at the moment of transplant. Subsequently, plants were watered every other day with a 50 ml aqueous solution of either 5 mM potassium silicate (SiO_2_K_2_O, Alfa Aesar^TM^) or 5 mM potassium chloride (KCl, EMD millipore) (pH. 6.8). Each plant received a total of 500 ml of either solution. KCl was used to replenish the amount of potassium in non-Si-supplemented control plants. Tomato and soybean plants were exposed to insect herbivory when their 5th leaf was fully extended. Maize (*Zea mays*) seeds of the herbivore susceptible genotype TX601 (inbred line) were obtained from W. P. Williams from Mississippi State University and the USDA-ARS, (Mississippi State, MS), and germinated in Promix potting soil (Premier Horticulture Quakertown, PA, USA). The seedlings were transplanted 10 d after germination into 3.78-l pots (C400 Nursery Supplies Inc. Chambersburg, PA, USA) containing Hagerstown loam soil and Promix potting soil mixed in relation 2:1. Each 3.78-l pot contained either 3 g of calcium silicate (CaSiO_3_, Alfa Aesar^TM^) or 3 g of lime (Ca (OH)_2_, Alfa Aesar^TM^) mixed with the soil. Lime was used to replenish the amount of calcium in control plants. The amounts of calcium silicate and lime applied to the soil mixture were calculated to raise the pH to 6.0–6.5 which is considered appropriate for maize growth (Unagwu et al., 2013). Maize plants were fertilized once with 3 g of Osmocote plus at the moment of transplant and were exposed to herbivory at their V7–V8 physiological stage (7–8 leaf collar). All plants were grown under glasshouse conditions (14 h light: 10 h dark) at the Pennsylvania State University, University Park, PA.

### Insects

Fall armyworm, *S. frugiperda* eggs were purchased from Benzon Research (Carlisle, PA, USA) and reared in laboratory at 25°C in 16:8 light:dark conditions. Larvae were reared individually in 30 ml cups (DART 100PC, Mason, MI, USA) containing ~5 ml of a wheat germ and casein-based artificial diet (Chippendale, 1970; Peiffer and Felton, 2005).

### Herbivore Treatment and Plant Defense Responses

Herbivore-induced plant defense responses were measured by quantifying the activity of plant defensive proteins, the expression of plant defensive genes, the concentration of total phenolics, and the number of trichomes in leaves. In tomato and soybean, we measured the enzymatic activities of three known herbivore-induced antinutritional proteins: polyphenol oxidase (PPO), peroxidase (POX), and trypsin protease inhibitor (trypsin PI). PPO and POX were measured at early (48 h in tomato, and 72 h in soybean) and late (15 d) time points following FAW herbivory, whereas trypsin PI was only measured at the early time points. In maize plants, the expression of a maize proteinase inhibitor gene (*mpi*) was measured 24 h (early time point) after FAW exposure. The concentration of total phenolics, the number of trichomes, and foliar Si content were quantified 15 d after FAW herbivory in tomato, soybean, and maize plants.

For early time point experiments, a set of plants supplemented and non-supplemented with Si were exposed to actively feeding last-instar FAW larvae enclosed in click cages (polypropylene with metallic micromesh screen, 23 mm diameter and 18 mm height) to standardize the amount of damage between treatments. FAW larvae were randomly assigned to the treatments and removed from plants after consuming the entire 415.48 mm^2^ of leaf tissue contained in the cage. The plant tissue surrounding the feeding sites was harvested 24, 48, and 72 h later for maize, tomato, and soybean, respectively. For late time point experiments, a separate set of Si-supplemented and non-Si-supplemented plants were exposed to heavy defoliation by FAW. Each tomato and soybean plant were exposed to three last-instar FAW larvae individually enclosed in cages (5.5 cm diameter, 1.5 cm high, 23.76 cm^2^ area) built with two plastic petri dish bottoms (60 × 15 mm, VWR, West Chester, PA, USA) held together with aluminum hair clips (Acevedo et al., 2018). These cages allowed larvae to feed on leaves attached to plants while preventing their spread in the greenhouse. The cages also helped standardize the amount of damage to about 90% per plant. Each maize plant was infested with one FAW larva placed at the whorl and allowed to eat freely for 3 days. Fifteen days after herbivore exposure, the new regrowth leaves were harvested for analyses. The fresh tissue excised from plants at early and late time points was immediately weighed, frozen in liquid nitrogen, and stored at −80°C for further analyses. We collected 50 mg of fresh tissue for enzymatic assays, 20 mg for quantification of phenolics, and 70 mg for RNA extractions. The remaining leaves were used for quantification of trichomes and for larval bioassays. Leaves from the late time point were also used for Si quantification; these were placed inside paper bags, dried at 60°C until constant weight, and ground to powder in a Udy cyclone mill (Thomas Scientific, Swedesboro, NJ. USA).

#### Activity of Defensive Enzymes

The activities of PPO and trypsin PI were measured following the procedure described by Chung and Felton (2011). Trypsin PI activity was calculated as described previously (Acevedo et al., 2017). POX activity assays followed a previously described protocol (Acevedo et al., 2017). The activity values from the enzymatic assays were normalized by the amount of protein (mg/ml) contained in each sample.

#### Concentration of Phenolics

The concentration of total phenolics in leaf samples was quantified following the Folin-Ciocalteu protocol (Ainsworth and Gillespie, 2007). The content of phenolics was expressed as mM of gallic acid equivalents per gram of fresh tissue.

#### Proteinase Inhibitor (*mpi*) Gene Expression

The procedures for RNA extraction, cDNA synthesis, and quantitative qPCR were carried out as previously described (Acevedo et al., 2017).

#### Density of Leaf Trichomes

Fifteen days after plants were exposed to insect herbivory, the newer fully expanded leaf was harvested from each plant to count the number of trichomes under a dissecting stereoscope (Olympus SZ30). In tomato, we counted the number of glandular trichomes type VI in an area of 0.24 mm^2^. In soybean, we registered the number of non-glandular type V trichomes in a 16 mm^2^ area. For maize, the number of non-glandular macro hairs or long trichomes was counted in an area of 2.84 cm^2^. For each plant species, two samples were taken per leaf, and their average number of trichomes was used for the statistical analyses. For imaging, leaf pieces of 0.24 mm^2^ were excised and immersed in a fixative solution (2.5% glutaraldehyde, 1.5% formaldehyde in 0.1M sodium cacodylate buffer pH. 7.4) for 48 h; subsequently, the samples were dehydrated through ethanol series and critical point dried with liquid CO_2_. The samples were then mounted in aluminum stubs with carbon tape and imaged in a Scanning Electron Microscope (SEM) at the Penn State Microscopy Facility (University Park, PA). The type VI trichomes in tomato are characterized for having four glandular cells connected to a short stalk, whereas the type V trichomes in soybean consist of a hollow long stalk cell with a multicellular base (Glas et al., 2012; Li et al., 2018). Maize macro hairs are long single cell stalks present in the adaxial epidermis of the leaves (Nelson et al., 2002). These trichome types are abundant, relatively easy to count and some have been associated with plant herbivore resistance (Glas et al., 2012).

### Si Quantification

Total Si was extracted with hydrofluoric acid (HF) and quantified with the molybdenum blue method reported by Diogo and Wydra (2007). Briefly, 30 milligrams of grounded tissue were placed in a 2 ml plastic tube to which 1 ml of extraction solution (1.5 M HF, 0.6 M HCl) was added; these tubes were then agitated overnight inside a fume hood. Samples were then centrifuged at 10,000 g for 10 min. Twenty microliters of the supernatant were transferred into a new 1.5 ml tube to which 230 μl of 3.2% boric acid (H_3_BO_3_) were added; tubes were agitated overnight. Subsequently, 250 μl of the color solution [1:1 mixture of 0.08 M H_2_SO_4_ and 20 mg/ml of (NH_4_)_6_ ${\text{Mo}}_{7}^{*}$ 4H_2_O] were added and incubated for 30 min. Then, 250 μl of 33 mg/ml of tartaric acid and 250 μl of 4 mg/ml of ascorbic acid were added. Lastly, 200 μl of the mixture were used to measure absorbance at 811 nm in a spectrophotometer (SpectraMax 190, Molecular Devices, San Jose, CA, USA). The amount of Si in the samples was determined using a blank-corrected standard curve constructed with different concentrations of a commercial Si [(NH_4_)_2_SiF_6_] standard (Cat. # 10M50-4F, High-Purity standards, Charleston, SC, USA).

#### Si Quantification in Maize Trichomes

To quantify the amount of Si deposited in maize macro hairs (trichomes), we excised leaves from either Si-supplemented or non-Si-supplemented plants. Those leaves were immediately taken to the lab and frozen in liquid nitrogen; long trichomes were extracted from frozen leaves using a scalpel (# 10). The trichomes were dried to constant weight for Si extraction and quantification following the procedure described in section Si Quantification. The deposition of Si in these trichomes was also analyzed with Energy Dispersive x-ray Spectroscopy (EDS) following the procedure below.

#### Energy Dispersive X-Ray Spectroscopy and Elemental Mapping

For microscopic observation of Si bodies, leaf pieces of 0.24 mm^2^ were excised and dehydrated in serially diluted ethanol solutions. These samples were critical point dried with liquid CO_2_ and mounted in aluminum stubs with carbon tape. Backscattered electron images (BSE), and EDS were carried out at the Penn State Materials Research Institute (University Park, PA) in an FEI Quanta 200 ESEM equipped with a 10 mm Si drift detector and the Aztec software version 2.3 (Oxford Instruments). The instrument was operated under low vacuum conditions of 70 Pa, at a voltage of 20 kV, and a working distance of 12.5 mm.

### Bioassays

#### Effect of Plant Si Supplementation and Insect Herbivory on Larval Weight Gain

FAW larval neonates were fed on detached leaves from either tomato, soybean or maize untreated plants until their second or third instar. Afterwards, larvae were weighed on a precision scale (Sartorius BP 61S, precision: 0.1 mg) to obtain their “initial weight” before being exposed to the corresponding treatments. Each larva was then individualized in 30 ml plastic cups (DART 100PC, Mason, MI, USA) containing detached leaves from either tomato, soybean or maize plants previously exposed to the different soil amendments (potassium silicate/potassium chloride or calcium silicate/lime) and herbivore treatments (FAW larval fed or undamaged control plants) described in the sections of Plants and Herbivore treatment and plant defense responses. Each cup had a 3 ml bottom layer of 2% agar to prevent dehydration of the leaves. Larvae were grown under laboratory conditions (25°C, 75% RH, and photoperiod of 16 h light: 8 dark) with constant food supply for 4–5 d, time at which their “final weight” was obtained. Larval weight gain was calculated as the difference between their final and their initial weights. Larvae were randomly assigned to each of the treatments in a complete randomized design.

#### Effect of Si on Larval Weight Gain and Mandible Wear

To test the effect of Si on FAW larval weight gain, freshly hatched neonates were individually placed inside 30 ml plastic cups (DART 100PC) containing a wheat germ and casein-based artificial diet (Chippendale, 1970; Peiffer and Felton, 2005) supplemented with either 0, 0.5, 1, 2, 5 or 10% Si dioxide (SiO_2_, SIGMA). Larvae were weighed 5 days later on a precision scale (Sartorius BP 61S). Thirty larvae were randomly assigned to each of the Si concentrations in a complete randomized design. To investigate the effect of Si on mandible wear, larvae from FAW were grown on non-Si-supplemented artificial diet (described in section Insects), for their first five instars. Newly molted six-instar larvae were then transferred to new cups containing the same type of diet supplemented with either 0, 2.5 or 5% Si dioxide (SiO_2_, SIGMA). After 3 d of feeding, the larval mandibles were dissected and placed in fixative solution (2.5% glutaraldehyde, 1.5% formaldehyde in 0.1 M sodium cacodylate buffer pH. 7.4). The samples were then washed with 0.1 M sodium cacodylate buffer, dehydrated through ethanol series and critical point dried with liquid CO_2_. The samples were mounted in aluminum stubs with carbon tape and imaged in an SEM at the Penn State Microscopy Facility.

#### Effect of Maize Trichomes on Larval Weight Gain

Leaves from maize plants (V7-V8) supplemented with Si, as indicated in section Plants, were detached and taken to the lab. The mid-portion of the leaves was cut out, the midvein was removed, and the non-glandular macro-hairs from one side (left or right from the midrib) of each leaf were excised using a razor blade under a stereoscope. The other side of the leaf was left intact. The leaf pieces with or without trichomes were used to feed FAW larvae. FAW neonates were grown on wheat germ diet (described in section Effect of Si on Larval Weight Gain and Mandible Wear) until they reached an average of 27 mg. Then, larvae (*n* = 20) were weighed and placed individually inside 30 ml plastic cups containing leaf pieces with or without trichomes. Two days later, the larvae were re-weighed to determine their weight gain. Each cup had a 3 ml bottom layer of 2% agar to prevent dehydration of the leaves. To determine if trichomes would break down in the larval gut, frass pellets from larvae fed on leaves with trichomes were placed in fixative solution, critical point dried, and photographed in an SEM.

### Experimental Design and Statistical Analyses

We used a two-factor factorial design that combined two Si treatments (Si treated and Si untreated) with two herbivore treatments (insect-fed and undamaged controls). The experimental units (either plants or insects) used in our experiments were randomly assigned to each of the treatments in a complete randomized design. We used two-way ANOVA for testing the effects of Si (Si supplemented vs. non-Si-supplemented plants) and herbivore treatments (larval fed vs. undamaged controls) on the activity of defensive enzymes (PPO, POX, and trypsin PI), the concentration of phenolics, the expression of *mpi*, the density of trichomes, larva weight gain, and the amount of Si in leaves. When the interaction factor was significant, a one-way ANOVA was used to elucidate the differences among treatment combinations. Differences between treatment means were obtained using either the Tukey's Honestly Significant Difference (HSD) test or the Tukey-Kramer test (for balance and unbalance replications, respectively) at alpha = 0.05; when needed, data were transformed to meet the assumptions of normality and equal variance before doing ANOVA. A two-sample *t*-test was used to identify differences in the amount of Si deposited in maize trichomes, and the effect of siliceous trichomes in larva weight gain. One-way ANOVA was used to test for differences in larva weight gain grown in diet supplemented with Si. ANOVAs and *t*-tests were conducted using the software Minitab 18 (Minitab Inc., State College, PA, USA). All graphs were generated in R version 3.6.6 (Foundation for Statistical Computing, Vienna, Austria).

## Results

### Si Supplementation and Insect Herbivory Induced Defense Responses in Si-Accumulator and Non-Si-Accumulator Plants

**Figure 1:**
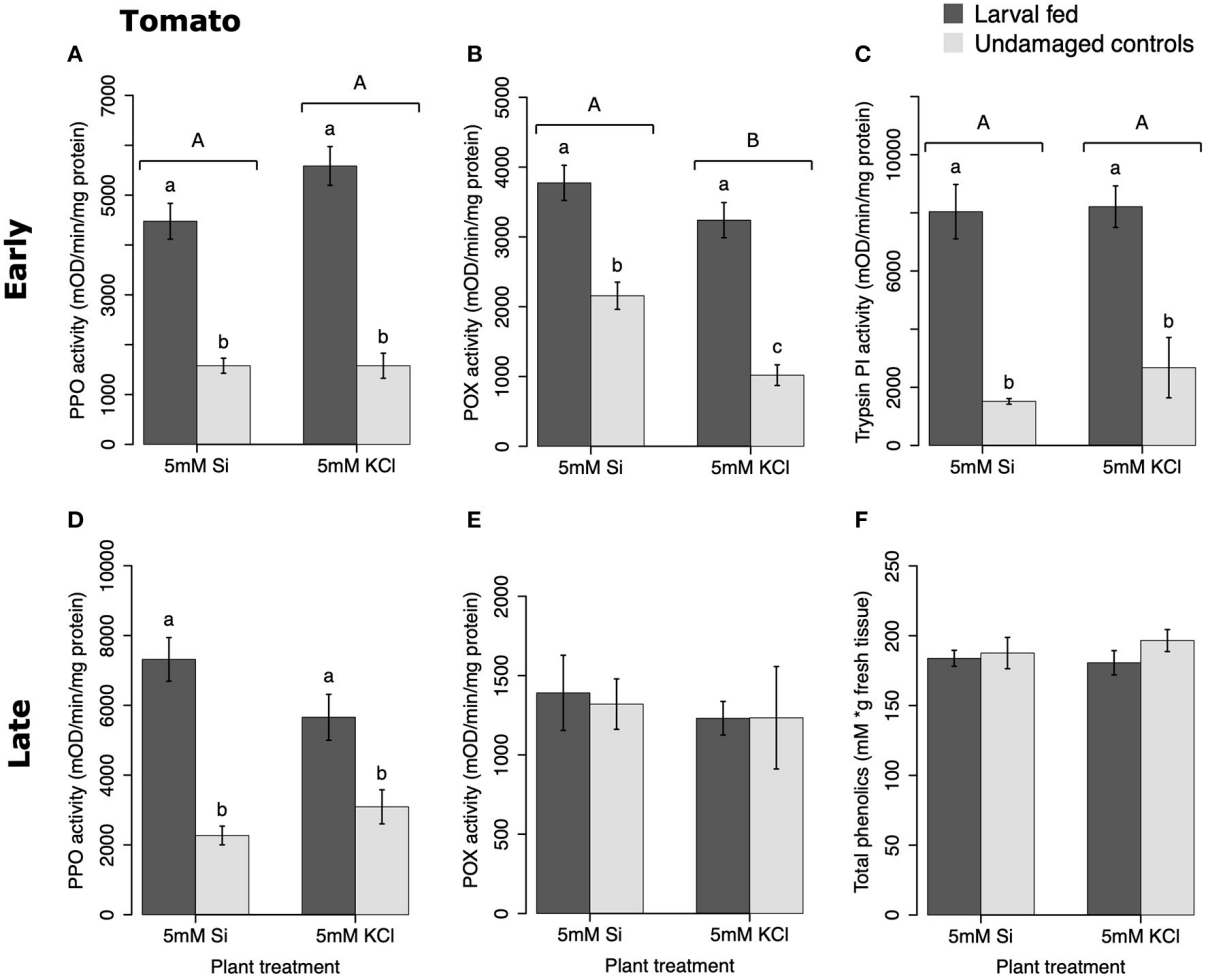
Herbivore-induced defense responses in tomato plants supplemented with either silicon (5 mM K_2_SiO_3_) or potassium chloride (5 mM KCl) and exposed to fall armyworm herbivory. Early and late defense responses are those measured 48 h and 15 d after herbivore exposure, respectively. The activity of plant defensive enzymes is represented in: **(A,D)** polyphenol oxidase (PPO), **(B,E)** peroxidase (POX), and **(C)** trypsin protease inhibitor (Trypsin PI). **(F)** The concentration of total phenolics is expressed as mM of gallic acid equivalents per gram of fresh tissue. Bar values are untransformed means ± SEM; different letters indicate significant differences obtained with ANOVA following Tukey tests at α = 0.05. Capital letters represent differences between the Si treatment and potassium chloride while lowercase letters indicate differences between treatments of herbivory and undamaged controls. In **(D)**, letters denote differences among treatment means obtained with the Tukey HSD test after one-way ANOVA.

**Figure 2:**
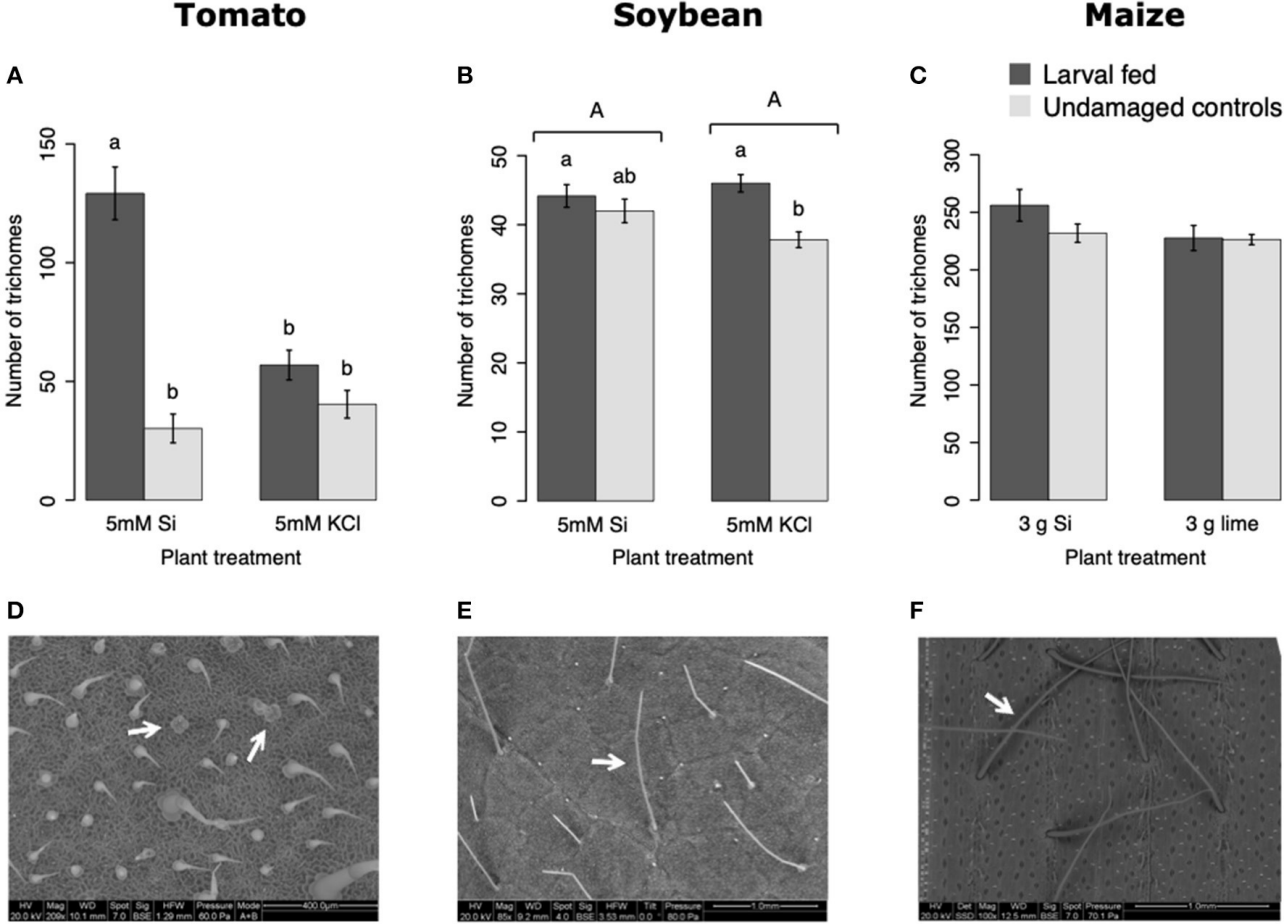
Number of trichomes in tomato **(A)**, soybean **(B)**, and maize **(C)** plants either supplemented with silicon or non-supplemented controls exposed and non-exposed to fall armyworm herbivory. Scanning electron images (SEM) of tomato **(D)**, soybean **(E)**, and maize **(F)** trichomes; white arrows indicate the type of trichomes counted in this study. Bar values are untransformed means ± SEM; different letters indicate significant differences obtained with ANOVA following Tukey tests at α = 0.05. Capital letters represent differences between Si treated and Si-untreated plants while lowercase letters indicate differences between treatments of herbivory and undamaged controls. In **(A)**, letters represent differences among treatment means obtained with the Tukey HSD test after one-way ANOVA.

In tomato, the enzymatic activities of polyphenol oxidase (PPO), peroxidase (POX), and trypsin protease inhibitor (trypsin PI) were greater in insect-fed plants compared with undamaged controls 48 h after larval exposure irrespective of the Si treatment (*P* < 0.05; Figure 1; Table 1). At this early time point, the activity of POX was higher in Si-supplemented plants compared with non-Si-supplemented controls (Figure 1B; Tukey HSD *t* = 3.72, *P* = 0.001). Fifteen days after larval damage, the activity of PPO was greater in plants exposed to herbivory compared with the undamaged controls [ANOVA *F*_(3, 36)_ = 19.06, *P* = 0.00], irrespective of the Si treatment (Figure 1D). The effects of the herbivory and Si treatments on PPO activity at 15 d were analyzed with a one-way ANOVA due to the significance of the interaction factor obtained in the two-way ANOVA (Table 1). At this late time point, the activity of POX and the concentration of total phenolics were not different among treatments (Figures 1E,F). The number of glandular trichomes in leaves was higher in plants supplemented with Si and exposed to insect herbivory compared with undamaged controls and non-Si-supplemented plants [Figure 2A; ANOVA *F*_(3, 20)_ = 34.23, *P* = 0.00]. The effects of the herbivory and Si treatments on the number of trichomes were analyzed with a one-way ANOVA due to the significance of the interaction factor obtained in the two-way ANOVA (Table 1).

**Table 1:** Two way ANOVA results of the effects of silicon (Si) (Si/KCl) and insect treatments (fed/undamaged controls) on the enzymatic activities of polyphenol oxidase (PPO), peroxidase (POX), and trypsin protease inhibitor (Trypsin PI) in tomato and soybean plants.

**Plant**	**Variable**	**Time**	**Effect**	**df (trt, error)**	**F**	***p*-value**	**Figures**
Tomato	PPO	Early	Si treatment	1,38	3.02	0.09	1A
			Insect treatment	1,38	117.19	0.000^*^	
			Interaction	1,38	3.02	0.09	
		Late	Si treatment	1,36	0.62	0.437	1D
			Insect treatment	1,36	51.14	0.000^*^	
			Interaction	1,36	5.42	0.026^*^	
	POX	Early	Si treatment	1,42	13.84	0.001^*^	1B
			Insect treatment	1,42	73	0.000^*^	
			Interaction	1,42	1.81	0.186	
		Late^a^	Si treatment	1,36	0.48	0.492	1E
			Insect treatment	1,36	0.17	0.680	
			Interaction	1,36	0.05	0.826	
	Trypsin PI	Early^a^	Si treatment	1,37	0.64	0.428	1C
			Insect treatment	1,37	67.62	0.000^*^	
			Interaction	1,37	0.32	0.573	
	Total phenolics	Late	Si treatment	1,36	0.11	0.738	1F
			Insect treatment	1,36	1.31	0.260	
			Interaction	1,36	0.50	0.484	
	Number of glandular trichomes	Late	Si treatment	1,20	16.5	0.001^*^	2A
			Insect treatment	1,20	57.09	0.000^*^	
			Interaction	1,20	29.10	0.000^*^	
	Leaf Si content	Late	Si treatment	1,23	50.95	0.000^*^	5A
			Insect treatment	1,23	1.7	0.205	
			Interaction	1,23	0.98	0.333	
	Larval weight gain	Early^a^	Si treatment	1,42	0.2	0.654	7A
			Insect treatment	1,42	401.66	0.000^*^	
			Interaction	1,42	0.83	0.367	
		Late	Si treatment	1,74	0.37	0.546	7D
			Insect treatment	1,74	18.15	0.000^*^	
			Interaction	1,74	0.13	0.723	
Soybean	PPO	Early	Si treatment	1,26	0.87	0.360	3A
			Insect treatment	1,26	0.85	0.365	
			Interaction	1,26	6.25	0.019	
		Late^b^	Si treatment	1,26	0.06	0.805	3D
			Insect treatment	1,26	0.18	0.672	
			Interaction	1,26	1.46	0.237	
	POX	Early	Si treatment	1,31	0.02	0.882	3B
			Insect treatment	1,31	19.74	0.000^*^	
			Interaction	1,31	0.63	0.434	
		Late	Si treatment	1,35	0.11	0.743	3E
			Insect treatment	1,35	4.47	0.042^*^	
			Interaction	1,35	0.00	0.962	
	Trypsin PI	Early	Si treatment	1,35	5.27	0.028^*^	3C
			Insect treatment	1,35	0.18	0.675	
			Interaction	1,35	12.14	0.001^*^	
	Total phenolics	Late	Si treatment	1,36	21.68	0.000^*^	3F
			Insect treatment	1,36	38.72	0.000^*^	
			Interaction	1,36	0.12	0.732	
	Number of trichomes	Late	Si treatment	1,20	0.64	0.434	2B
			Insect treatment	1,20	12.53	0.002^*^	
			Interaction	1,20	4.22	0.053	
	Leaf Si content	Late	Si treatment	1,24	234	0.000^*^	5B
			Insect treatment	1,24	24.99	0.000^*^	
			Interaction	1,24	2.13	0.157	
	Larval weight gain	Early	Si treatment	1,36	4.18	0.048^*^	7B
			Insect treatment	1,36	15.14	0.000^*^	
			Interaction	1,36	0.42	0.519	
		Late	Si treatment	1,103	0.31	0.576	7E
			Insect treatment	1,103	12.62	0.001^*^	
			Interaction	1,103	1.04	0.310	
Maize	mpi gene expression	Early^b^	Si treatment	1,20	2.21	0.153	4A
			Insect treatment	1,20	129.47	0.000^*^	
			Interaction	1,20	3.15	0.091	
	Total phenolics	Late^a^	Si treatment	1,32	0.69	0.411	4B
			Insect treatment	1,32	18.17	0.000^*^	
			Interaction	1,32	3.76	0.061	
	Number of long macro hairs	Late	Si treatment	1,19	2.53	0.128	2C
			Insect treatment	1,19	1.14	0.298	
			Interaction	1,19	1.43	0.246	
	Leaf Si content	Late	Si treatment	1,24	211.84	0.000^*^	5C
			Insect treatment	1,24	5.50	0.028^*^	
			Interaction	1,24	0.36	0.556	
	Larval weight gain	Early^a^	Si treatment	1,46	53.28	0.000^*^	7C
			Insect treatment	1,46	16.31	0.000^*^	
			Interaction	1,46	0.17	0.682	
		Late	Si treatment	1,32	7.51	0.010^*^	7F
			Insect treatment	1,32	0.08	0.783	
			Interaction	1,32	0.45	0.509	

The table also depicts the results of the effects of Si and insect treatments on the relative expression of a maize proteinase inhibitor gen (mpi), and on the concentration of total phenolics, the number of trichomes, leaf Si content, and larval weight gain in tomato, soybean, and maize plants. The effects of Si and insect treatments on plant defensive compounds and larval weight gain were tested at different time points for each plant species: 48 h (early response) and 15 d after treatment (late response) in tomato; 72 h (early response) and 15 d after treatment (late response) in soybean; and 24 h (early response) and 15 d after treatment (late response) in maize plants. Asterisks denote significant p-values

(*) *at α = 0.05. df, degrees of freedom; trt, treatment*.

a *Squared root transformed*.

b *Log transformed*.

**Figure 3:**
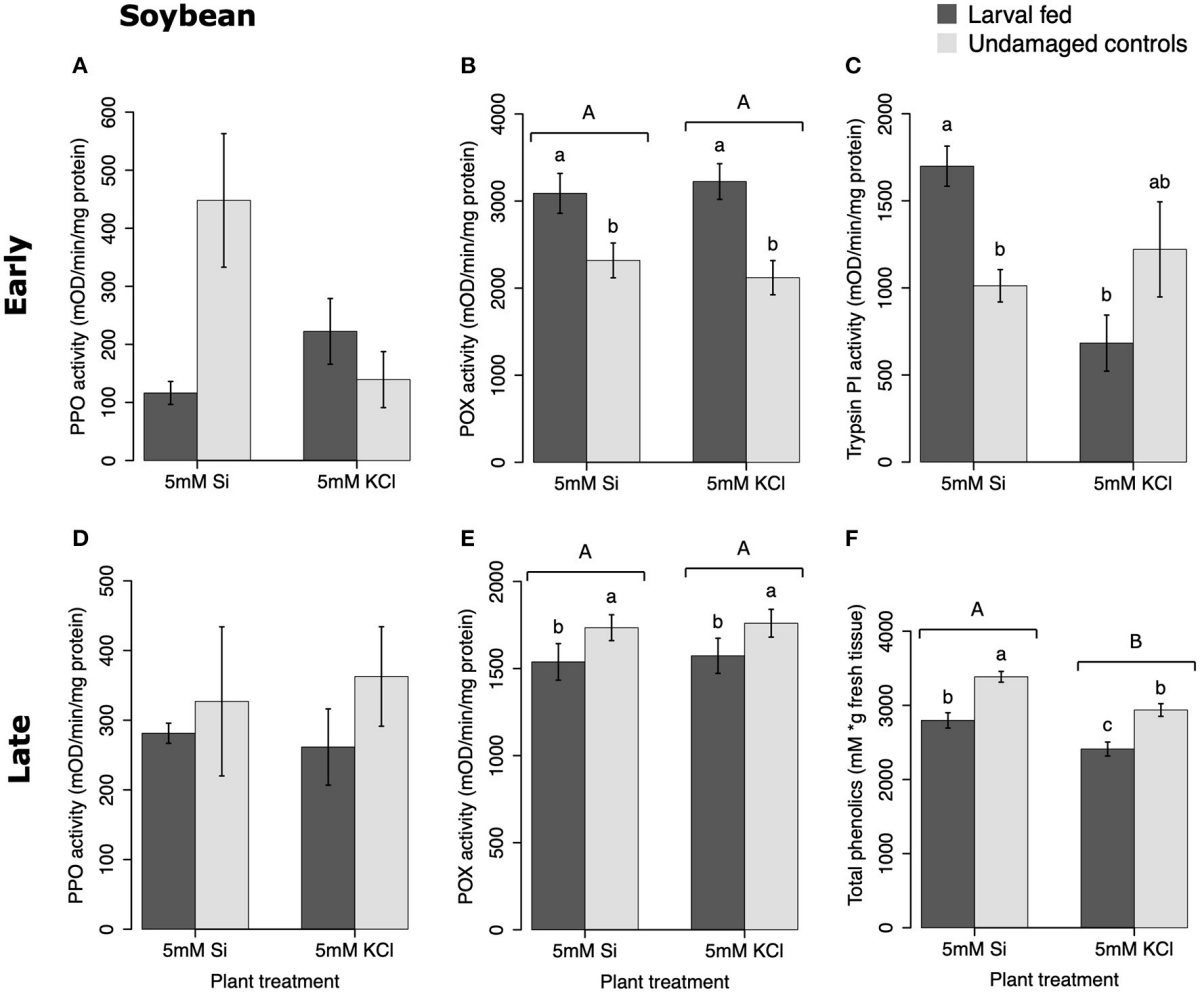
Herbivore-induced defense responses in soybean plants supplemented with either silicon (5 mM K_2_SiO_3_) or potassium chloride (5 mM KCl) and exposed to fall armyworm herbivory. Early and late defense responses are those measured 72 h and 15 d after herbivore exposure, respectively. The activity of plant defensive enzymes is represented in: **(A,D)** polyphenol oxidase (PPO), **(B,E)** peroxidase (POX), and **(C)** trypsin protease inhibitor (Trypsin PI). **(F)** The concentration of total phenolics is expressed as mM of gallic acid equivalents per gram of fresh tissue. Bar values are untransformed means ± SEM; different letters indicate significant differences obtained with ANOVA following Tukey tests at α = 0.05. Capital letters represent differences between the Si treatment and potassium chloride while lowercase letters indicate differences between treatments of herbivory and undamaged controls. In **(C)**, letters represent differences among treatment means obtained with the Tukey-Kramer test after one-way ANOVA.

In soybean, the enzymatic activities of POX and Trypsin PI, and the concentration of total phenolics were affected by insect feeding and Si treatment (Figure 3). The activity of POX was higher in both Si-supplemented and non-Si-supplemented plants fed on by FAW within 72 h of larval exposure compared with undamaged controls (Figure 3B). However, 15 d after insect treatment, the activity of POX had an opposite trend being greater in undamaged controls when compared with insect-fed plants (Tukey-Kramer *t* = −2.11, *P* = 0.042); no effect of the Si treatment was found at this late time point (Figure 3E). The activity of Trypsin PI was higher in Si-supplemented plants exposed to herbivory compared with Si-supplemented undamaged controls and non-Si supplemented larval fed plants [Figure 3C; ANOVA *F*_(3, 35)_ = 5.77, *P* = 0.003]. The effects of the herbivory and Si treatments on Trypsin PI activity were analyzed with a one-way ANOVA due to the significance of the interaction factor obtained in the two-way ANOVA (Table 1). Fifteen days after insect treatment, the concentration of total phenolics was higher in plants supplemented with Si compared with non-Si-supplemented controls; their concentration was also greater in undamaged controls compared with larval fed plants (Figure 3F; Table 1). The activity of PPO was not different among treatments in either early or late time points (Figures 3A,D). The number of trichomes was higher in soybean plants exposed to herbivory, but there was no effect of the Si-treatment (Table 1; Figure 2B).

**Figure 4:**
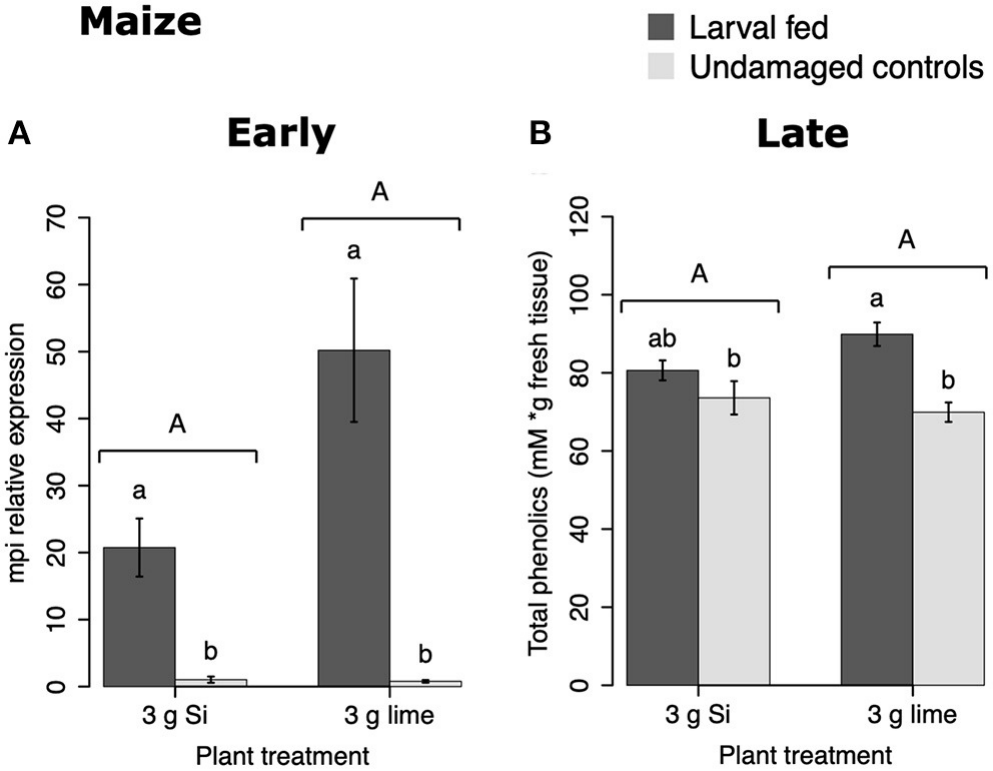
Herbivore-induced defense responses in maize plants supplemented with either silicon (3 g Ca_2_SiO_4_) or lime [3 g Ca (OH) _2_] and exposed to fall armyworm herbivory. Early and late defense responses are those measured 24 h and 15 d after herbivore exposure, respectively. **(A)** Relative expression of a maize proteinase inhibitor gene (mpi), and **(B)** concentration of total phenolics is expressed as mM of gallic acid equivalents per gram of fresh tissue. Bar values are untransformed means ± SEM; different letters indicate significant differences obtained with ANOVA following Tukey tests at α = 0.05. Capital letters represent differences between Si treated and Si-untreated plants while lowercase letters indicate differences between treatments of herbivory and undamaged controls.

In maize, the gene expression of *maize proteinase inhibitor (mpi)* was greater in insect-fed plants 24 h after herbivory compared with undamaged controls (Figure 4A). Likewise, the concentration of total phenolics in maize was higher in insect-fed plants 15 d after herbivory compared with intact controls (Figure 4B; Table 1). There was no effect of Si supplementation on either *mpi* expression or total phenolics (Figure 4). The density of long trichomes in maize plants was not affected by the treatments of herbivory or Si supplementation (Table 1; Figure 2C).

### Si Supplementation and Herbivory Affected Leaf Si Accumulation

**Figure 5:**
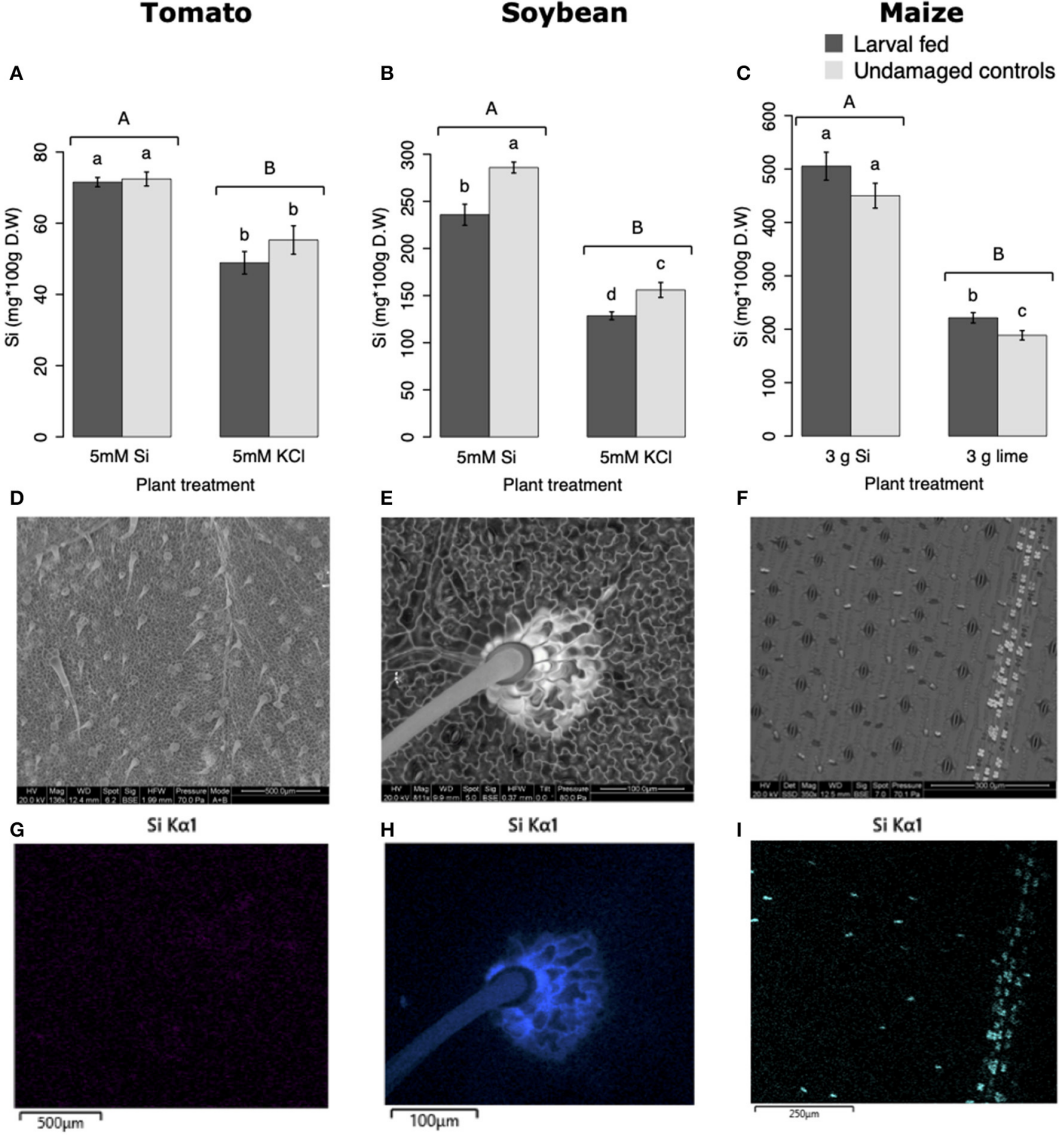
Silicon accumulation in: **(A)** tomato, **(B)** soybean, and **(C)** maize leaves; plants were either supplemented with silicon or non-supplemented controls exposed and non-exposed to fall armyworm herbivory. Bar values are untransformed means ± SEM; different letters indicate significant differences obtained with ANOVA following Tukey tests at α = 0.05. Capital letters represent differences between Si treated and Si-untreated plants while lowercase letters indicate differences between treatments of herbivory and undamaged controls. Backscattered electron images (BSE) of tomato **(D)**, soybean **(E)**, and maize **(F)** leaves show mineral deposition in leaf epidermis and trichomes (white structures). Si distribution in tomato **(G)**, soybean **(H)**, and maize **(I)** leaves obtained with energy-dispersive X-ray spectroscopy (EDS).

**Figure 6:**
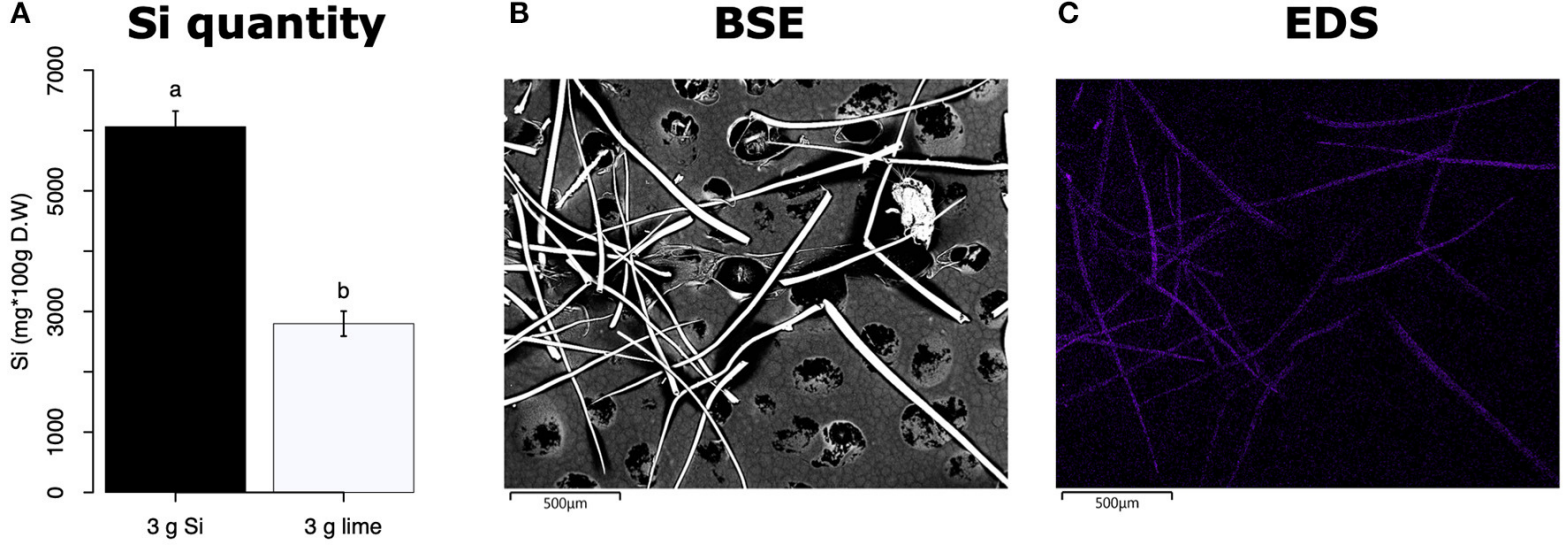
**(A)** Si content in maize trichomes extracted from plants supplemented with either Si (3 g Ca_2_SiO_4_) or lime [3 g Ca (OH)_2_]. **(B)** Backscattered electron (BSE) images of long macro hairs extracted from maize leaves. **(C)** Si distribution in maize trichomes obtained with energy-dispersive X-ray spectroscopy (EDS), the purple coloration represents accumulation of Si.

All Si-supplemented plants accumulated greater amount of this element in their tissues than non-Si-supplemented controls (Figure 5). Tomato plants treated with Si had on average 0.72 ± 0.022 (95% CI) mg/g (dry tissue) of leaf Si content, whereas non-Si treated plants contained 0.52 ± 0.05 mg/g of this element. The foliar Si concentrations in soybean were 2.6 ± 0.18 and 1.42 ± 0.11 mg/g in Si-supplemented and non-Si-supplemented plants, respectively. In maize, there was 4.77 ± 0.36 mg/g of Si in Si-treated plants, and 2.05 ± 0.15 mg/g in Si-untreated controls. Herbivory also influenced the accumulation of Si in soybean and maize plants, but not in tomato (Figure 5). Soybean plants fed on by fall armyworm larvae accumulated less Si than undamaged controls (Figure 5B). In contrast, fall armyworm herbivory induced higher accumulation of Si in maize plants non-supplemented with Si but growing in soil with Si content (Figure 5C). Si-supplemented tomato plants had higher Si content than non-Si supplemented controls (Figure 5A), but we were unable to detect Si structures in leaves through BSE or EDS (Figures 5D,G). Si accumulation in soybean was found in trichomes and leaf epidermal compartments at the base of those trichomes (Figures 5E,H). Maize plants accumulated Si in trichomes and silica cells (Figures 5F,I, 6). Although the density of long trichomes in maize leaves was not affected by Si-supplementation or insect herbivory (Figure 2C), the amount of Si contained in those trichomes was higher in Si-supplemented plants compared to non-Si-supplemented controls (Figure 6A; *t* = −9.85, *P* = 0.000, *n* = 4). In maize, Si deposition occurred in the whole trichome from base to tip (Figures 6B,C).

### Bioassays

#### Plant Si Supplementation and Former Insect Herbivory Affected Larval Weight Gain

**Figure 7:**
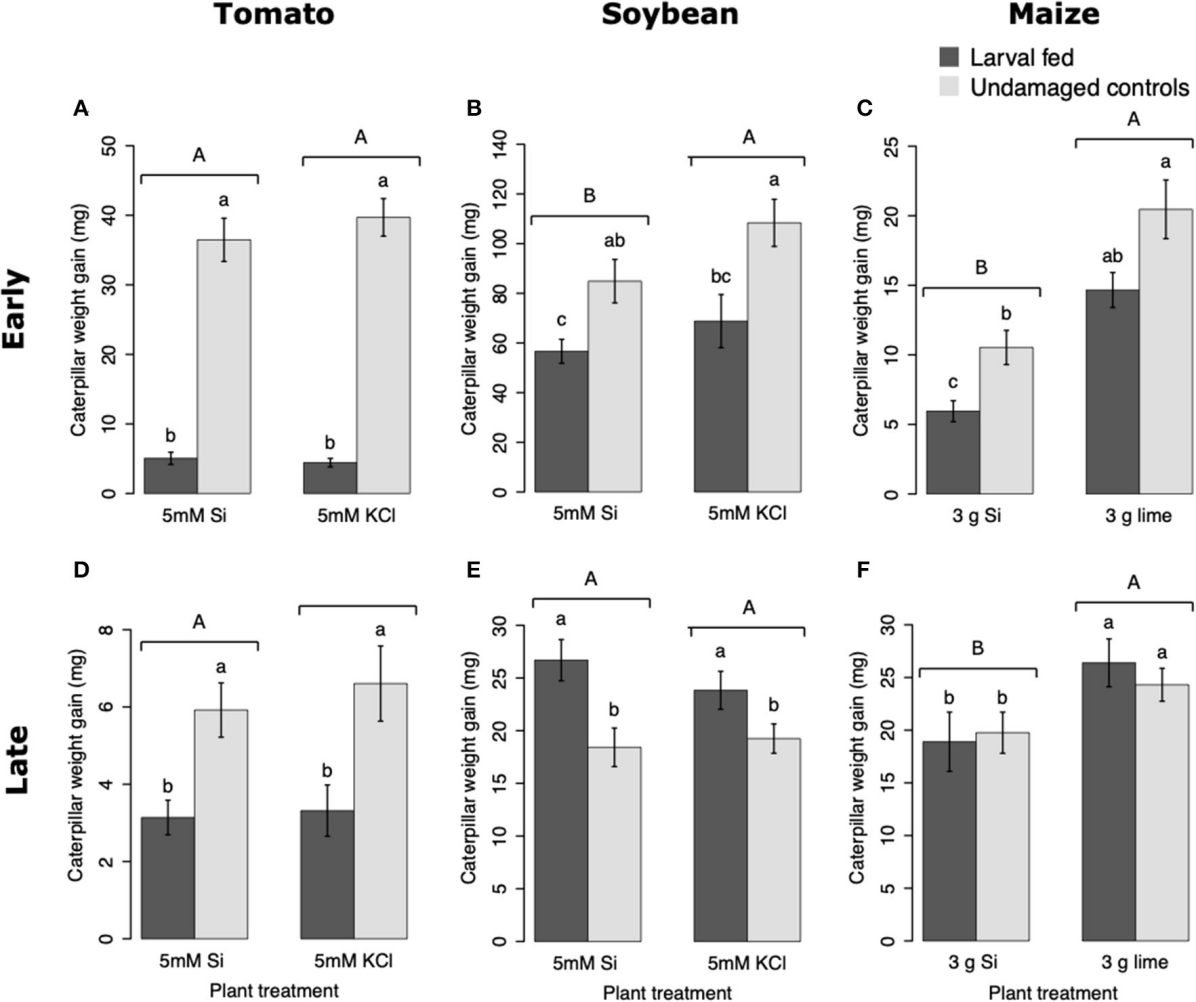
Weight gain of fall armyworm (FAW) larvae fed on tomato **(A,D)**, soybean **(B,E)**, and maize **(C,F)** plants either supplemented or non-supplemented with Si with or without previous exposure to herbivory. Early and late, correspond to different time points at which the effects of Si plant supplementation and previous insect herbivory were tested on FAW larval weight gain. For tomato, early represents 48 h, and late represents 15 d after plants were treated with FAW larvae. In Soybean, early and late refers to 72 h and 15 d after herbivore treatment, respectively. For maize, early denotes 24 h whereas late indicates 15 d after herbivory. Bar values are untransformed means ± SEM; different letters indicate significant differences obtained with ANOVA following Tukey tests at α = 0.05. Capital letters represent differences between Si treated and Si-untreated plants while lowercase letters indicate differences between treatments of herbivory and undamaged controls.

Larvae fed on tomato plants previously exposed to herbivory gained less weight at the early and late time points than those fed on undamaged controls; plant Si-Supplementation did not affect larva weight gain (Table 1; Figures 7A,D). In soybean, former herbivory influenced larval weight gain at early and late time points, but Si-supplementation only had an effect at the early time point (Table 1). Larvae fed on soybean leaves detached from plants exposed to herbivory 72 h earlier gained less weight than those fed on undamaged plants (Figure 7B). Conversely, young FAW larvae gained more weight when fed on leaves detached from soybean plants exposed to herbivory 15 d earlier than those fed on undamaged control plants (Figure 7E). In maize, the Si treatment resulted in lower larval weight gain at both early and late time points, whereas previous herbivory reduced larval weight gain only at the early time point (24 h) (Table 1; Figures 7C,F).

#### Si-Supplemented Artificial Diet Induced Larval Mandible Wear but Did Not Affect Weight Gain

**Figure 8:**
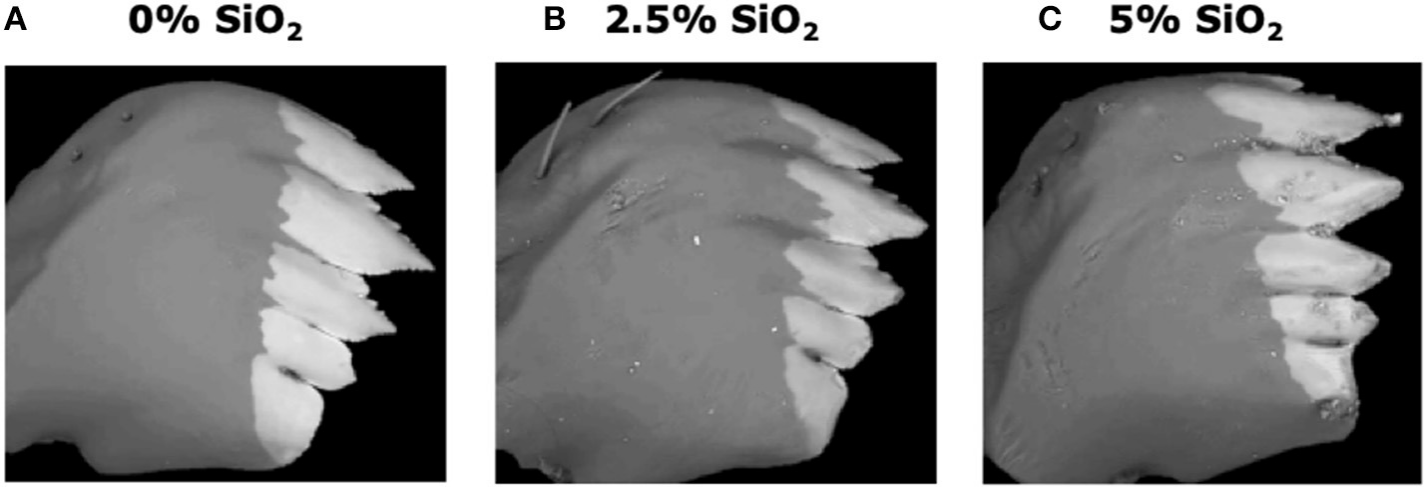
Backscattered electron images of six-instar fall armyworm (FAW) mandibles extracted from larvae fed on artificial diet containing various amounts of silicon dioxide: **(A)** 0% SiO_2_, **(B)** 2.5% SiO_2_, and **(C)** 5% of SiO_2_. White areas represent the distribution of mineral accumulation (mainly zinc).

Si-containing diets enhanced larval mandible wear. FAW larvae fed on the artificial diet supplemented with SiO_2_ had greater mandible wear than larvae fed on the diet without Si. Greater mandible wear was observed in larvae fed on the diet supplemented with 5% SiO2 than in those fed with 2.5% of Si (Figure 8). Contrarily, Si-supplemented diet had no effect on larval weight gain [*F*_(5, 172)_ = 0.77, *P* = 0.570; *n* = 28–30].

#### Siliceous Maize Macro-Hairs Reduced FAW Larval Weight

**Figure 9:**
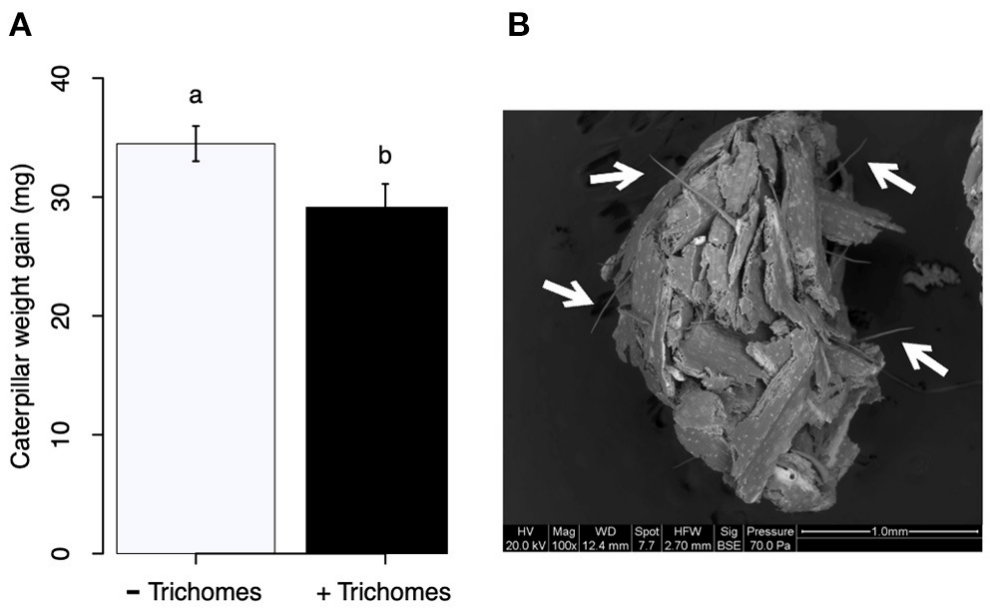
**(A)** Weight gain of fall armyworm (FAW) larvae fed on maize leaves without (–Trichomes) or with (+Trichomes) siliceous macro hairs. **(B)** Frass pellet from FAW larvae fed on leaves with macro hairs. White arrows point to trichomes that appear to be intact after their passage through the larval gut.

FAW larvae fed on maize leaves with macro hairs gained less weight than those fed on leaves from which trichomes were removed (Figure 9A; *t* = 2.18, *df* = 35, *P* = 0,036, *n* = 20). Trichomes did not break down in the larval gut; rather they appear to have been excreted almost intact (Figure 9B).

## Discussion

In this study, we investigated if Si supplementation would increase herbivore resistance in crop plants with differential ability to accumulate this element. Our results show that herbivory and Si supply augmented the levels of some plant biochemical and physical defenses at early and late time points in a species-specific manner. Additionally, Si supplementation increased leaf Si content in all plants. Former herbivory affected FAW larval weight gain in all plants tested, and the Si treatment further reduced weight gain of larvae fed on Si accumulator plants. Notably, our results strongly suggest that non-glandular trichomes are important reservoirs of Si in maize and may increase plant resistance to chewing herbivores.

### Si Supplementation and Insect Herbivory Influence Plant Defense Responses

**Figure 10:**
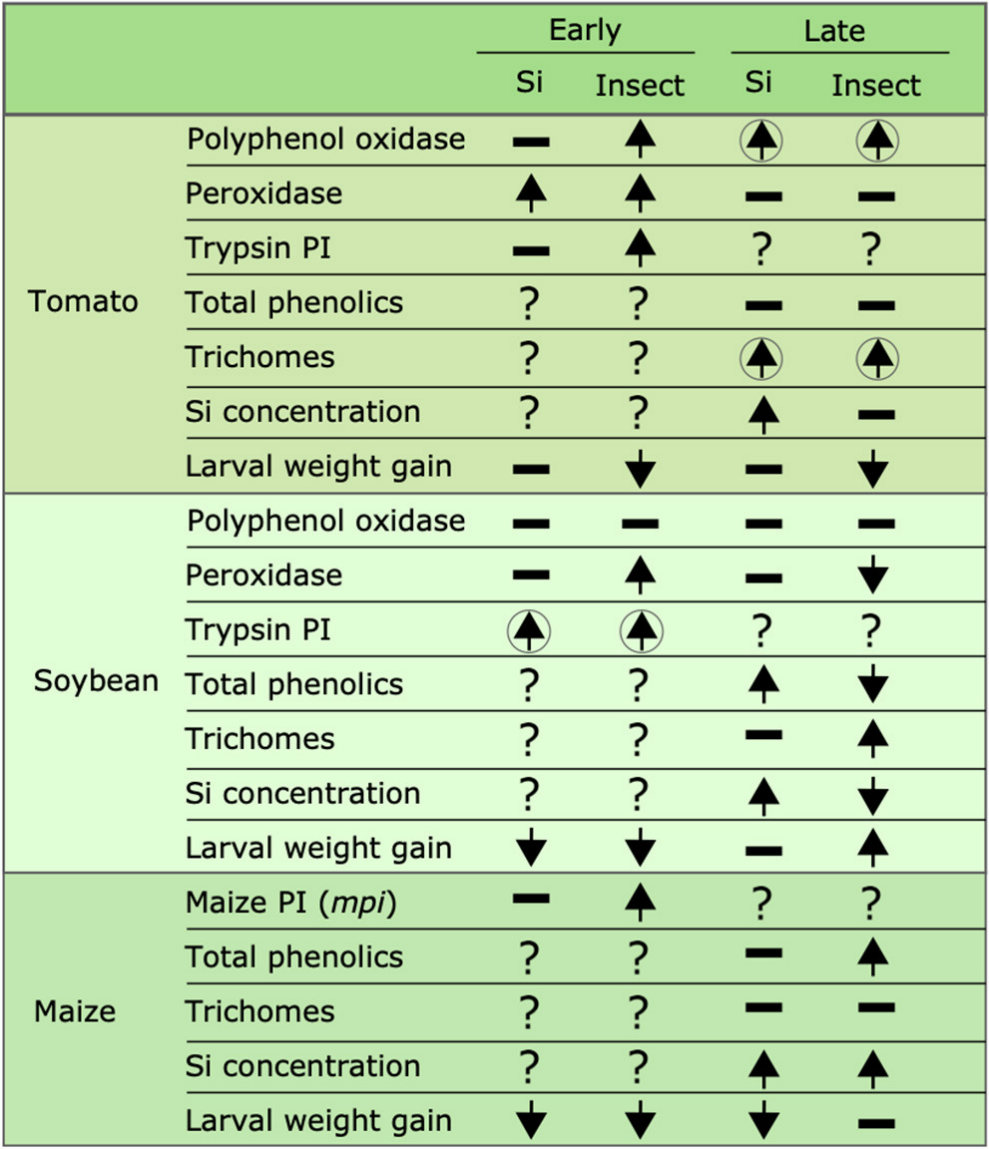
Summary of the effects of Si supplementation and insect herbivory on plant defense responses and larval weight gain at early and late time points in tomato, soybean, and maize. Upward arrows represent a significant increase, downward arrows represent significant reduction, hyphens represent no significant effects, and question marks indicate that the effect was not tested. Arrows enclosed in circles indicate that the combination of both Si supplementation and insect herbivory had a significant effect. Early effects were measured 24, 48, and 72 hrs after treatment in maize, soybean, and tomato, respectively; late effects were measured 15 d after treatment in all plant species.

In tomato plants, FAW herbivory increased PPO, POX, and trypsin PI activities within 48 h of larval exposure (Figure 10). The Si treatment further enhanced POX activity at this early time point (Figure 1B). PPO was still upregulated 15 d after herbivore exposure, but POX and total phenolics were not different among treatments at the late time point. Furthermore, the herbivore treatment combined with Si supplementation increased the number of glandular trichomes in tomato leaves 15 d after herbivory (Figure 2A). The effect of Si on herbivore-induced defenses in tomato has been scarcely studied, but induction of plant resistance has been reported (Faraone et al., 2020). There is also evidence that Si enhances resistance of tomato plants against pathogens. For example, Diogo and Wydra (2007) found less incidence of the bacterial wilt disease, *Ralstonia solanacearum* in tomato plants treated with Si. This enhancement of resistance appears to be correlated with higher activities of the defensive proteins POX, PPO, phenylalanine ammonia lyase (PAL), and lipoxygenase as well as higher total soluble phenolics (Jiang et al., 2019). Likewise, the Si treatment reduced the severity of soil-borne diseases in tomato caused by *Fusarium oxysporum* (Huang et al., 2011). Previous studies have demonstrated that insect feeding induces the number of glandular trichomes in tomato plants (Tian et al., 2012); interestingly, our results indicate that the number of trichomes is enhanced by Si supplementation in the presence of herbivory. These results suggest that Si supplementation enhances some biochemical and physical defenses in tomato despite the low capability of this species to accumulate Si.

In soybean, insect feeding induced greater production of trichomes irrespective of the Si treatment (Figure 2B; Table 1). The combination of Si and FAW herbivory induced higher activity of trypsin PI at 72 h compared with undamaged Si-supplemented controls and herbivore-treated plants not supplemented with Si (Figure 3C). The Si treatment induced greater accumulation of total phenolics than those found in non-Si-supplemented plants 15 d after herbivore exposure (Figure 3F; Table 1). In contrast to our results, a previous study reported a decrease in total phenolics in non-insect infested soybean plants treated with Si (Ferreira et al., 2011). The discrepancy of these results may be explained by differences in herbivore treatments and sampling time points between studies or genetic variation among soybean cultivars. FAW herbivory induced higher activity of POX 72 h after treatment. But 15 d later, the POX activity levels and the concentration of total phenolics were downregulated in herbivore-treated plants with respect to untreated controls. Shifts in the activity patterns of defensive enzymes in response to herbivory have been previously reported in soybean. Locateli et al. (2019) found higher activity of PAL in soybean plants 96 h after treatment with *Bemisia tabaci*, but at 168 h the PAL activity was lower than that of untreated controls. Speculatively, it may be possible that herbivore-derived effectors change the initial plant response over time. Si supplementation appears to enhance some biochemical defense responses in this plant species.

In maize, FAW feeding induced early (*mpi* expression) and late defense responses (total phenolics). But Si addition did not affect the expression of *mpi*, the concentration of total phenolics or the density of leaf macro hairs in maize plants (Figures 2C, 4, 10). Si-mediated increase in maize resistance to herbivores has been reported previously (Sétamou et al., 1993; Boer et al., 2019; Moise et al., 2019) but the mechanisms have not been elucidated. Further RNA-seq or quantitative proteomic studies may help reveal the means by which Si enhances herbivore resistance in maize.

### Si Supplementation Enhances Si Accumulation in Plants

The Si treatment increased foliar Si content in Si-accumulators and non-Si accumulator plants regardless of herbivory. Si supplementation in tomato, soybean, and maize increased the foliar content of this element by 38.4, 83, and 132.7%, correspondingly, with respect to the Si content in non-Si-supplemented plants (Figure 5). Increase in shoot Si content in response to Si supply has been previously reported in these plant species (Huang et al., 2011; Boer et al., 2019; Johnson et al., 2020b). Some studies have also shown that herbivory induces Si deposition in Si-accumulator plants (Massey et al., 2007; Johnson et al., 2020b); in our studies, there was a slight significant increase of Si in maize plants exposed to FAW herbivory but not in tomato or soybean. The Si content was higher in maize leaves followed by soybean and tomato which can be explained by the differential ability of these plants to uptake Si (Deshmukh et al., 2015; Coskun et al., 2019). Maize and soybean contain functional influx and efflux transporters, whereas tomato only contains Si influx, but not functional Si efflux transporters (Deshmukh et al., 2015; Sun et al., 2020). Variable Si accumulation in maize and soybean plants may also be associated with differences in the density and possibly size of silica cells and non-glandular trichomes. Si-containing cells are present in row arrangements as well as spread out in the leaf epidermis of maize and other grasses, whereas in soybean, silica was found in the basal cells of non-glandular trichomes (Figures 5E–I). Moreover, the maize genotype used in this study contained three times more macro-hairs per squared mm than soybean (Figures 2B,C). Notably, non-glandular trichomes appear to be important structures for Si deposition, leaf macro-hairs extracted from Si-supplemented maize plants had 116.5% more Si than those extracted from non-Si supplemented controls (Figure 6). Accumulation of Si in the tips of trichomes has been observed in grasses, but there are no previous reports of their actual Si concentration (de Melo et al., 2010; Andama et al., 2020). Si may increase the rigidity of trichomes and physically harm the digestive system of chewing herbivores. Trichomes are often ingested along with leaf tissue and excreted almost intact in larval frass (Figure 9).

### Si Supplementation and Insect Herbivory Influence FAW Larval Weight Gain

Larval weight gain was affected by the Si and herbivore treatments in a specific way for each plant species. FAW larvae gained less weight when fed on tomato, soybean, and maize plants previously exposed to herbivory (Figure 7). In tomato, reduction in larval weight gain at early and late time points was exclusively associated with former herbivory and was negatively correlated with higher activity levels of PPO. In soybean, former herbivory and the Si treatment reduced larval weight gain at the early time point, but there was no correlation with the activity of defensive enzymes measured in this study. Contrarily, at the late time point, FAW larvae gained more weight when fed on soybean plants previously exposed to herbivory; this was negatively correlated with POX activity and the concentration of total phenolics. Si supplementation to soybean plants has been shown to reduce the growth of *Helicoverpa punctigera* larvae within 6 days of herbivore exposure, and to increase mortality of whitefly nymphs fed on intact plants (Ferreira et al., 2011; Johnson et al., 2020b). Therefore, the potential of Si to enhance plant protection may differ with time and depend on specific herbivore-plant interactions. In Maize, there was a decrease in larval weight gain associated with previous herbivory and Si treatments at the early time point. But the reduction in larval weight at the late time point was only present in Si-treated maize plants which may be negatively correlated with the presence of tougher trichomes. Si, by itself, did not decrease herbivore weight, but trichomes with higher Si content appear to affect larval growth (Figure 9). Indeed, other studies have shown that leaf macro hairs reduce the relative growth rate of chewing herbivores (Johnson et al., 2020a). Si is also likely to affect other herbivore parameters not measured in this study. For example, Si treatment of maize plants reduced fecundity of FAW moths (Alvarenga et al., 2017), reduced growth rate of the stemborer *Busseola fusca* (Juma et al., 2015), and increased mortality of the lepidopteran larvae *Pseudaletia unipuncta* and *Sesamia calamistis* (Sétamou et al., 1993; Moise et al., 2019). Herbivory and Si treatments are likely to trigger various plant responses, including changes in gene expression, protein abundance, and nutritional quality. Although our ability to draw conclusions are limited to the plant responses tested in this study, the topic deserves further investigation using more comprehensive omics techniques.

### Mechanisms of Si-Mediated Plant Resistance Against Insect Herbivores

Si increases resistance to herbivores through physical and biochemical mechanisms (Alhousari and Greger, 2018). The role that physical depositions of Si (e.g., increase in toughness and abrasiveness) play on plant protection against herbivores is widely accepted, but the mechanisms by which Si mediates induction of biochemical defenses is currently under debate. Biochemical compounds that confer resistance to herbivory are regulated by plant hormones such as JA, SA, and ET (War et al., 2012). Some studies have reported an increase in JA in response to Si treatment, but others have found lower levels of this hormone (Hall et al., 2019). Due to the inability of Si to interact biochemically with the cell machinery, Coskun et al. (2019) proposed the so-called “apoplastic obstruction hypothesis.” In addition to the mechanical protection conferred by silica deposition in plant tissues, this model proposes that Si protection against herbivores is attributed to the physical movement obstruction of herbivore effectors within cells mediated by Si deposition in the cell apoplast. Although compelling, this argument contradicts numerous reports of systemic induction of plant biochemical responses triggered by herbivores in Si-treated plants. Alternatively, Si physical deposition may increase plant stress and result in upregulation of defensive pathways (Ye et al., 2013; Hall et al., 2019). In this case, upregulation of plant defenses would also be found in plants not exposed to herbivory. In line with the physical role of Si in mediating plant protection, Hall et al. (2019) proposed that Si-accumulator plants with constitutive antiherbivore defenses exhibit lower JA levels because of their existing physical protection. Our results indicate that Si enhances physical plant defenses in the form of Si accumulation in leaves and trichomes, and augment some JA-dependent biochemical defenses. The proposed models aim to promote further investigation to identify the underlying mechanism by which Si confer plant resistance to biotic and abiotic stresses (Coskun et al., 2019; Hall et al., 2019). These mechanisms may vary based on the plant' ability to accumulate Si and the nature of the stress.

### Conclusions

Our results show that FAW herbivory induces production of biochemical and physical defenses in tomato, soybean, and maize plants. The Si treatment enhanced some of these defenses, but resistance to herbivory measured as a reduction in larva weight gain was only observed in soybean at the early time point and in maize at early and late time points (Figure 10). Si alone did not reduce larva weight gain, but Si deposited in non-glandular trichomes did reduce weight gain. This study and others (Andama et al., 2020; Johnson et al., 2020a) emphasize the potential role of silicified trichomes in increasing plant resistance against chewing herbivores. We conclude that Si offers transient resistance to FAW in soybean and a longer duration of resistance in maize. Further studies are needed to assess the effect of transient and long-term defense responses in plant fitness under single and multiple herbivore events. Si supply appears to be a promising strategy in management programs of chewing herbivores in Si-accumulator plants.

## Data Availability Statement

The raw data supporting the conclusions of this article will be made available by the authors, without undue reservation.

## Author Contributions

FA and GF designed the study. FA and MP conducted the experiments. SR and C-WT helped with bioassays and plant treatments. FA analyzed the data and wrote the manuscript. All authors read, contributed to revisions, and approved the manuscript.

## Conflict of Interest

The authors declare that the research was conducted in the absence of any commercial or financial relationships that could be construed as a potential conflict of interest.
